# Levodopa exposure and nigral neuroinflammation in parkinsonian disorders: A postmortem study of 63 cases

**DOI:** 10.1038/s41598-025-23376-2

**Published:** 2025-11-11

**Authors:** Emmilotta A. Backman, Laura Luntamo, Tero Vahlberg, Maria Gardberg, Valtteri Kaasinen

**Affiliations:** 1 Clinical Neurosciences, University of Turku, Turku, Finland; 2 Neurocenter, Turku University Hospital, Turku, Finland; 3 Department of Biostatistics, University of Turku, Turku University Hospital, Turku, Finland; 4 Tyks Laboratories, Pathology, Institute of Biomedicine, Turku University Hospital, University of Turku, Turku, Finland

**Keywords:** Levodopa, Parkinson’s disease, Progressive supranuclear palsy, Multiple system atrophy, Neuroinflammation, Postmortem, Diseases, Neurology, Neuroscience

## Abstract

Levodopa remains the cornerstone of treatment in Parkinson’s disease (PD), but its long-term impact on neuroinflammation remains unclear, particularly in multiple system atrophy (MSA) and progressive supranuclear palsy (PSP), where levodopa efficacy is limited. This study examined whether chronic levodopa exposure is associated with neuroinflammation or dopaminergic neuronal loss in end-stage PD, MSA, and PSP. Postmortem midbrain tissue from 63 neuropathologically confirmed cases (PD: *n* = 38; PSP: *n* = 13; MSA: *n* = 12) was analyzed. Immunohistochemistry was used to quantify tyrosine hydroxylase positive (TH+) neurons in the substantia nigra pars compacta (SNc), along with T lymphocyte (CD3+, CD4+, CD8+) infiltration and microglial density (Iba1 expression). Levodopa exposure was estimated using three models and analyzed in relation to neuropathological markers, adjusting for age at death, sex, disease duration, and Hoehn & Yahr stage. Results. No significant associations were observed between levodopa exposure and TH + neuronal density or T cell infiltration or microglial density. For example, in PD, mean daily levodopa dose was not associated with CD3 + T cell density (β = 2.06 × 10⁻⁵, 95%CI: −0.001 to 0.001, *p* = 0.96). Chronic levodopa use was not associated with persistent neuroinflammation or dopaminergic neuronal loss at end-stage disease, suggesting no long-term immune-mediated toxicity in the substantia nigra.

**Supplementary Information:**

The online version contains supplementary material available at 10.1038/s41598-025-23376-2.

## Introduction

Levodopa remains the gold-standard symptomatic treatment for Parkinson’s disease (PD) by effectively restoring dopaminergic deficits in the basal ganglia^1–3^. Despite its therapeutic success in PD, its efficacy is markedly heterogeneous when applied to atypical parkinsonian disorders such as progressive supranuclear palsy (PSP) and multiple system atrophy (MSA), both of which present with overlapping motor symptoms but different underlying pathophysiology and clinical trajectories^4,5^.

While levodopa provides significant symptomatic benefit in PD, its long-term use is complicated by the emergence of motor fluctuations, including wearing-off phenomena and levodopa-induced dyskinesias, which significantly impact quality of life and complicate clinical management^6,7^. These observations, combined with laboratory evidence suggesting potential levodopa-induced oxidative stress and toxicity^8–11^, have raised concerns about its long-term safety and its role in disease progression. However, a post-mortem brain analysis concluded that levodopa use does not accelerate dopaminergic disease progression in PD nor induce neurotoxicity^12^, and an animal study on MSA indicated that even high doses of levodopa do not induce neurotoxicity^13^. On the other hand, some studies have proposed that levodopa may even have neuroprotective effects by maintaining dopamine levels to support neuronal function, potentially slowing disease progression^14,15^. The question of levodopa’s impact becomes even more relevant in atypical parkinsonian disorders, where clinical responses to levodopa are variable. Despite often limited benefit in PSP and MSA, levodopa remains a standard choice also in these disorders for both diagnostic and symptomatic purposes^5,16^.

Neuroinflammation is increasingly recognized as a key driver of neurodegeneration in parkinsonian syndromes, including PD, PSP, and MSA^17,18^. While the interplay between neuroinflammation and levodopa treatment has been hypothesized—particularly regarding its role in levodopa-induced dyskinesias^19^—it has not been rigorously investigated, using postmortem neuropathological analysis or in vivo neuroimaging data.

In this study, we aim to address this issue by evaluating the relationship between levodopa exposure, nigral neuronal survival, and neuroinflammation in PD, PSP, and MSA. By utilizing postmortem brain tissue analysis, we seek to determine whether chronic levodopa treatment influences neuronal loss or neuroinflammation in the substantia nigra pars compacta (SNc) — a region central to the motor dysfunction.

## Methods

### Subjects

The study cohort included neuropathologically confirmed cases of PD, PSP, and MSA examined between 2002 and 2021 at the Department of Pathology, Turku University Hospital, Finland. All neuropathological samples had been formalin-fixed and paraffin-embedded (FFPE). As quality control, each SN sample was re-examined histologically by an experienced neuropathologist before inclusion. Samples showing autolytic changes or not obtained from the level of the third cranial nerve were excluded. To address potential degradation, we recorded the postmortem interval (PMI) from death to autopsy, which did not differ significantly across groups.

#### Ethical approval

was obtained from the local ethics committee, and the study was conducted in accordance with the Declaration of Helsinki. The current study cohort was derived from a previous investigation, which included a total of 67 patients^18^; however, four patients were excluded from the present analysis due to incomplete medication data. A small number of patients (PD: *n* = 1, MSA: *n* = 4, PSP: *n* = 6) had no documented levodopa exposure. These were retained to ensure full representation of the exposure spectrum and served as internal controls.

All PD patients had received a clinically accurate diagnosis of PD prior to death, based on their clinical presentation and response to levodopa treatment, in line with the UK Brain Bank criteria. In the PSP group, six patients had been accurately diagnosed with PSP antemortem, three were categorized as having undetermined parkinsonism, two had been misdiagnosed with PD, and two had received a clinical diagnosis of corticobasal syndrome. Among the MSA patients, six had been correctly diagnosed with MSA during life, while two were initially misdiagnosed with PD, three were classified as having undetermined parkinsonism, and one received a clinical diagnosis of motor neuron disease.

Clinical and medical histories were systematically reviewed using the hospital’s electronic medical record system and archived patient charts. Demographic data, including sex, age at death, and disease duration - defined as the interval between the reported onset of motor symptoms to death – were collected. Information on various clinical symptoms emerging during the disease course was recorded, as described previously^18,20,21^. Additionally, relevant clinical parameters were collected where available, including the occurrence of dyskinesias, the last documented levodopa equivalent daily dose (LEDD) and Hoehn and Yahr (HY) stage. Descriptive data on motor and non-motor features, including rigidity, bradykinesia, resting tremor, and cognitive problems, were also documented. A structured data collection approach, incorporating standardized checklists, was employed to ensure consistency and completeness.

## Neuropathology

The neuropathological methods used to assess tyrosine hydroxylase-positive (TH+) dopaminergic cells in the SNc have been previously described in detail^21^. Briefly, midbrain FFPE samples were sectioned at 8 μm for TH staining and at 3.5 μm for other staining procedures. These sections were then subjected to Luxol fast blue or Substance P immunohistochemistry to delineate the borders of the SNc. TH immunohistochemistry was used to identify dopaminergic neurons, while immunohistochemistry for CD3, CD4, and CD8 was used to detect T lymphocytes, T helper cells, and cytotoxic T cells, respectively, as described^18^. All neurons stained positive for TH were counted, regardless of staining intensity. Based on prior data using this same staining protocol^22^, only 7.8% of neuromelanin-positive SNc neurons lacked TH immunoreactivity at end stage, suggesting that most surviving dopaminergic neurons are captured by this method. Microglial cells were identified using Iba1 immunohistochemistry^18^. The Crus cerebri, a neighboring white matter tract with low baseline microglial density, was used as a control region to compare Iba1 expression. Although not directly comparable in tissue type, its consistent presence in all samples and proximity to the SNc allowed for standardized anatomical sampling across cases. The slides were scanned with a Pannoramic P250 Flash slide scanner equipped with a Zeiss Plan-Apochromat 20×/0.8 NA objective, a CIS VCC-F52U25CL camera (Vital Vision Technology Pte Ltd., Singapore), and control software version 1.18.2. The images were analyzed using CaseViewer software version 2.4.0.119028 (3D HISTECH Ltd., Budapest, Hungary). TH-positive neurons in the SNc were manually and systematically counted at high magnification. The entire SNc area was evaluated in each case, from single sections. To address potential overcounting errors that can arise from analyzing large objects in two-dimensional sections relative to section thickness, neuron counts were corrected using the Abercrombie method^23^. To evaluate T-cell numbers and densities and Iba1 expression, QuPath version 0.5.0 (https://qupath.github.io), an open-source software for bioimaging analysis, was used^24^. The detailed methods for using the automatic cell detection tool, which we employed to detect T cells and microglial cells, have been previously described^18^.

## Levodopa data

Comprehensive levodopa usage data were systematically extracted from patient charts to capture treatment patterns across the entire disease course for each patient. Given the dynamic nature of levodopa dosing over time and the presence of missing data for certain time points in some patients, three distinct metrics of levodopa exposure were calculated to provide an assessment of cumulative and time-specific levodopa use.

The calculated indicators of levodopa exposure were as follows:

1. **Cumulative lifetime dose of levodopa (formula 1)**: This measure was adapted from Parkkinen et al. and O’Sullivan et al^12,14^., and was calculated using the following equation:

(LED [mg] at 1 year after commencement × 365) + ½ (maximum LED + LED at 1 year after commencement × 365) × (interval from 1 year after commencement to reaching maximum LED in years) + (maximum LED × 365) × (interval from reaching maximum LED to death in years).

LED = levodopa daily dose.

**2. Cumulative lifetime dose of levodopa not taking into account the multipliers (formula 2)**: This measure considers only the average daily levodopa dose and the duration of the disease, calculated using the following equation:

([LED at 1 year after commencement + maximum LED + LED at death]/3) x disease duration in years.

**3. The mean levodopa dose (formula 3)**: This measure considers only levodopa daily doses at different time points, calculated using the following equation:

(LED at 1 year after commencement + maximum LED + LED at death)/3.

## Statistical analyses

All statistical analyses were performed using SPSS Statistics 29 for Macintosh (IBM Corp., Armonk, NY, USA). Data distribution was assessed through visual inspection of histograms and the Kolmogorov‒Smirnov test to determine normality. Comparison of continuous variables across the three diagnostic groups (PD, PSP, and MSA) were conducted using the Kruskal‒Wallis test, with Bonferroni adjusted post hoc analyses to account for multiple comparisons. To examine associations between levodopa exposure and neuropathological markers in PD patients, an analysis of covariance (ANCOVA) was performed, applying logarithmic transformations to cell counts and densities to normalize distributions. Covariates were specified separately for each analysis and included age at death, sex, HY stage, disease duration and/or diagnostic group as appropriate. β coefficients with 95% confidence intervals were calculated for logarithmic values. Due to the smaller sample sizes in the PSP and MSA cohorts, partial Spearman correlation analyses were used to assess the relationship between levodopa exposure and neuropathological markers in these groups. Disease duration was controlled as a covariate. To account for multiple comparisons, a p-value of < 0.01 was considered statistically significant for analyses involving neuropathological markers. For baseline demographic, clinical, and neuropathological characteristics, a p-value of < 0.05 was considered statistically significant.

## Results

### Study participants

The study cohort comprised 63 individuals with neuropathologically confirmed diagnoses: 38 with PD, 13 with PSP, and 12 with MSA. The primary demographic and clinical characteristics of the participants are summarized in Table 1. Levodopa treatment was administered at some point during the disease course in 97% of PD patients (*n* = 37/38), 67% of MSA patients (*n* = 8/12), and 54% of PSP patients (*n* = 7/13). Dyskinesia was documented in four PD patients, with one patient experiencing symptoms in the left extremities, another in the face and right extremities, while the specific location was not reported in two cases. The median daily levodopa dose at the onset of the first dyskinesia was 600 mg [IQR = 263], and the median time from the beginning of levodopa medication to the onset of dyskinesia was 4.8 years [IQR = 5.1]. Analysis of levodopa exposure metrics revealed positive correlations between lifetime cumulative levodopa dose, mean levodopa dose, and levodopa dose at death (Supplementary Table 1), indicating internal consistency across different measures of levodopa exposure.

**Table 1:** Group differences in baseline demographic, clinical, and neuropathological characteristics. Data are presented as median [IQR] for continuous variables and n for categorical variables.

Variable group	Variable		Group			P value
			PD	PSP	MSA	
**Demographics**	Number of patients		38	13	12	-
	Age at death (years)		80.3 [9.2]*^†^	71.3 [11.6]	70.3 [22.6]	< 0.001
	Sex (m/f)		30/8^†^ (79%)	8/5 (62%)	5/7 (42%)	0.045
**Clinical characteristics**	Disease duration from motor symptom onset to death (years)		9.8 [7.0] ^†^	7.0 [8.3]	4.3 [4.6]	0.011
	HY stage (last before death)		4 [2]*^†^	5 [1]	5 [0]	< 0.001
	LEDD at death (mg)		525 [438]*	0 [380]	496 [1056]	0.008
	Motor phenotype (tremor/no tremor)		24/7* (77%)	5/8 (38%)	6/6 (50%)	0.031
	Asymmetry of motor symptoms (asymmetrical/symmetrical)		26/0*^†^(100%)	5/6^†^ (45%)	0/10 (0%)	< 0.001
**Levodopa**	Lifetime levodopa dose (formula 1) (kg)		0.68 [1.10]*	0.11 [0.49]	0.27 [1.09]	0.021
	Lifetime levodopa dose (formula 2) (kg)		1.2 [1.9]*	0.049 [1.01]	0.35 [0.93]	0.005
	Daily mean levodopa dose (formula 3) (mg)		467 [206]*	200 [325]	367 [646]	0.004
	Daily levodopa dose at death (mg)		500 [300]*	0 [350]	350 [800]	0.008
	Duration of levodopa use (years)		6.6 [7.1]	5.5 [7.1]	3.1 [4.4]	ns
	Duration of dopamine agonist use (years)		7.2 [7.6]	6.6 [7.6]	3.7 [4.0]	ns
**Nigral neuropathology**	SNc TH + neuron count (n)		61.6 [31.6]*^†^	29.7 [32.1]	24.9 [10.7]	< 0.001
	SNc area (mm^2^)		33.7 [7.5] ^†^	27.1 [11.5]	26.1 [8.3]	0.003
	SNc TH + neuron density (corrected n/mm^2^)		2.14 [1.46] ^†^	1.23 [0.74]	0.90 [0.41]	0.003
	SNc asymmetry index (%)^A^		78.5 [28.2] ^†^	67.3 [27.4]	63.6 [41.2]	0.045
	Brain weight (g)		1460 [187]	1372 [85]	1455 [233]	ns
	Death to autopsy delay (days)		6 [5]	3 [7]	4 [4]	ns
**Neuroinflammation markers**	CD3+	Count (n)	204 [133]*	375 [326] ^†^	234 [100]	0.009
		Area (mm^2^)	33.5 [8.4] ^†^	27.6 [11.1]	26.1 [7.8]	0.009
		Density (n/mm^2^)	6.67 [3.16]*	15.5 [9.2]	8.73 [4.07]	0.007
	CD4+	Count (n)	139 [191]*	265 [324] ^†^	195 [137]	0.002
		Area (mm^2^)	32.8 [6.4] ^†^	29.6 [9.2]	27.0 [9.7]	0.028
		Density (n/mm^2^)	4.08 [5.73]*	7.77 [11.2]	8.03 [4.66]	< 0.001
	CD8+	Count (n)	101 [108]	210 [192] ^†^	72 [129]	0.008
		Area (mm^2^)	33.7 [7.1] ^†^	29.1 [11.5]	26.5 [5.5]	0.002
		Density (n/mm^2^)	3.10 [3.50]*	8.87 [7.25] ^†^	3.03 [4.65]	0.013
	Microglia	SNc Iba1 expression (%)	2.07 [1.71] ^†^	1.14 [1.89]	1.15 [1.86]	0.016
		Crus cerebri Iba1 expression (%)	2.39 [1.10]	2.73 [2.93]	2.71 [2.17]	ns

* Significantly different (*p* < 0.05) compared to PSP after Bonferroni correction, ^†^ Significantly different (*p* < 0.05) compared to MSA after Bonferroni correction.

PD = Parkinson’s disease, PSP = progressive supranuclear palsy, MSA = multiple system atrophy, HY = Hoehn and Yahr, LEDD = levodopa equivalent daily dose, SNc = substantia nigra pars compacta, TH = tyrosine hydroxylase, ns = non-significant. P values are from the Kruskal‒Wallis test, chi‒square test or Fisher’s exact test. ^A^ (lower/higher side neuron count) × 100.

## PD patients

**Fig. 1:**
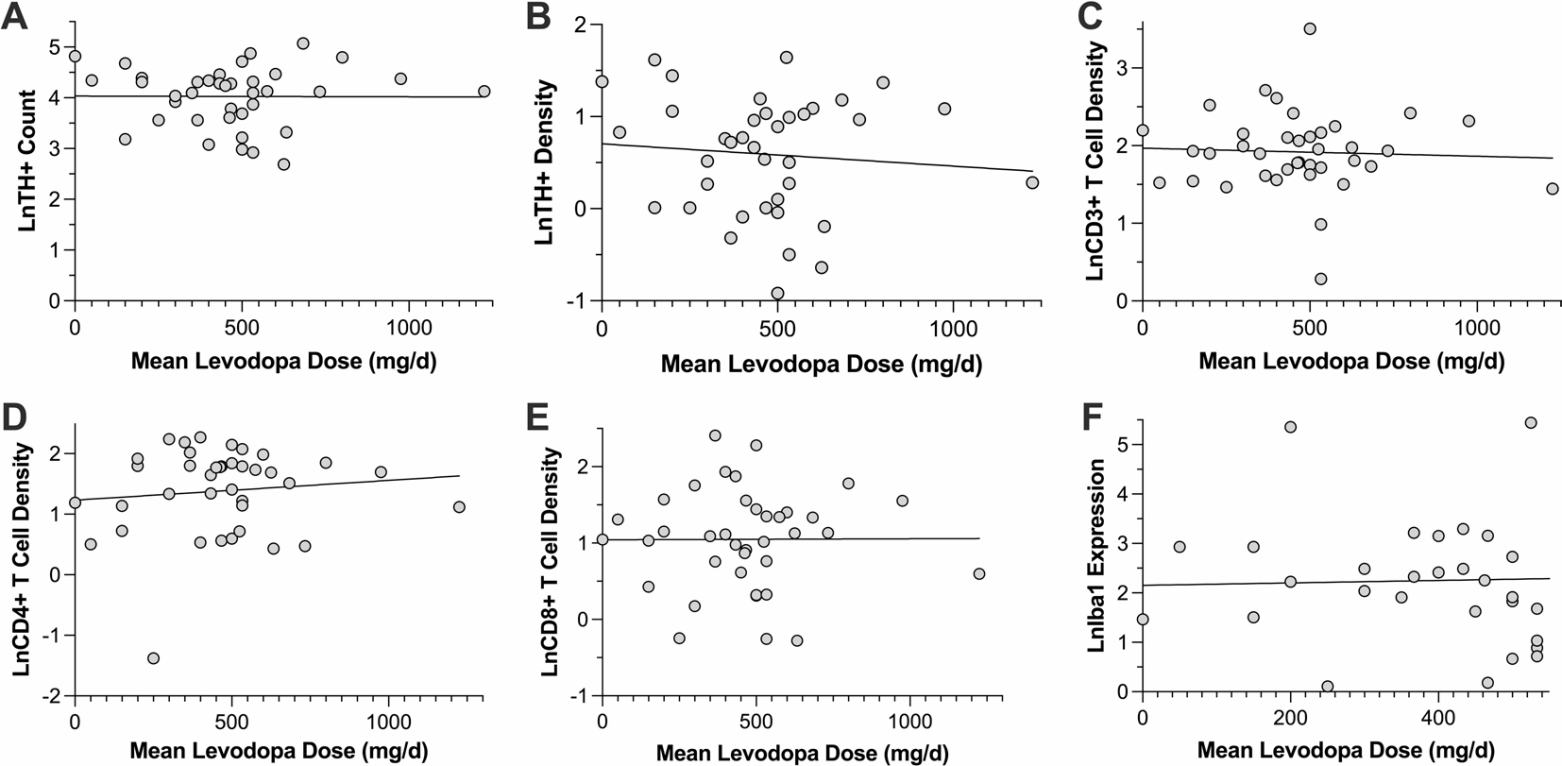
Scatter plots illustrating the relationships between mean daily levodopa dose over the disease course and neuropathological measures in the substantia nigra pars compacta (SNc) in PD patients. (**A**) Tyrosine hydroxylase-positive (TH+) neuron count vs. mean daily levodopa dose, (**B**) TH + neuron density vs. mean daily levodopa dose, (**C**) CD3 + T-cell density vs. mean daily levodopa dose, (**D**) CD4 + T-cell density vs. mean daily levodopa dose, (**E**) CD8 + T-cell density vs. mean daily levodopa dose, and (**F**) Iba1 expression in the SNc vs. mean daily levodopa dose. No significant associations were observed between mean daily levodopa dose and any neuropathological measure. Similar results were found for lifetime cumulative levodopa dose and levodopa dose at death.

**Fig. 2:**
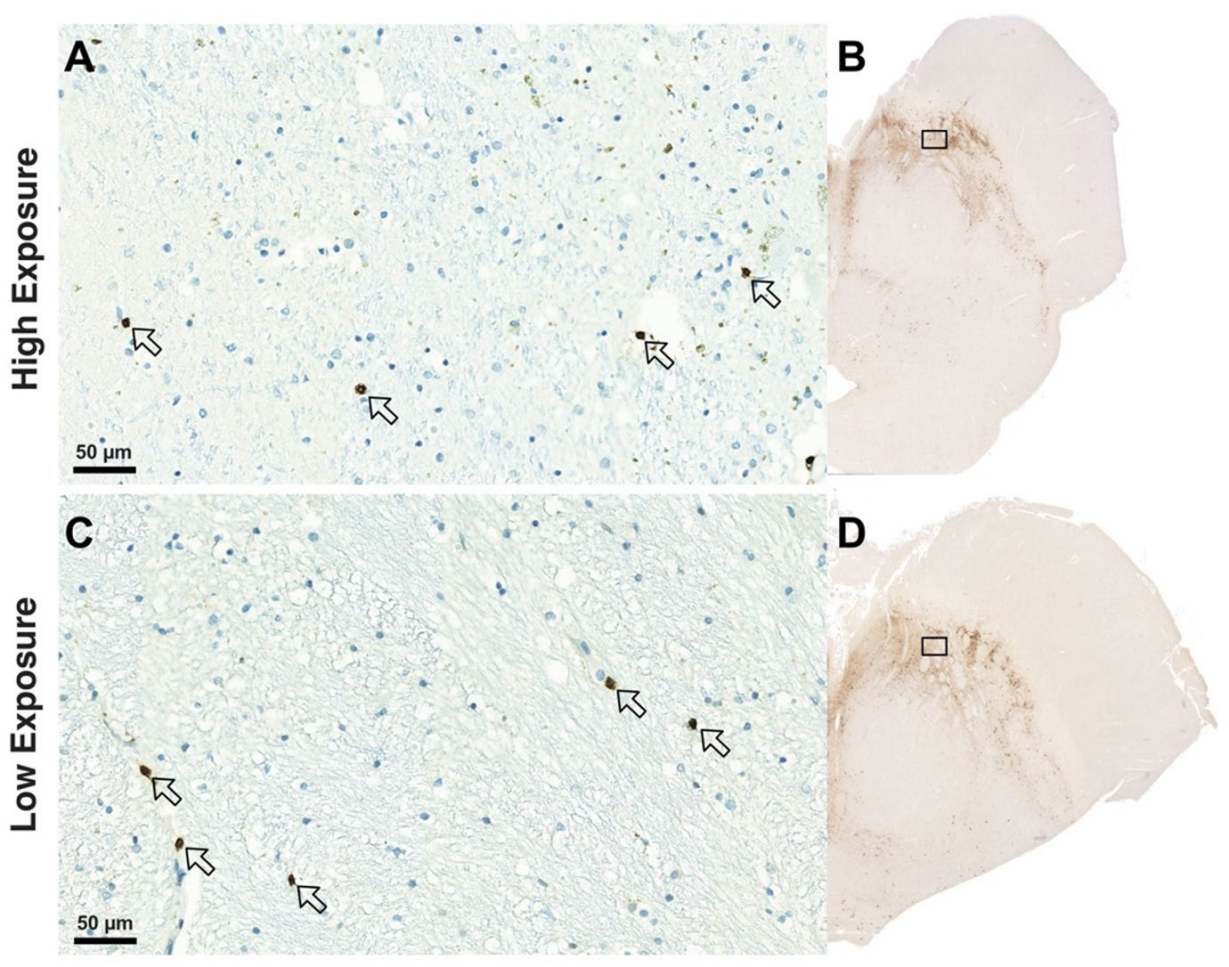
Representative images of the substantia nigra pars compacta (SNc) from two PD patients with markedly different lifetime levodopa exposures (formula 2), but comparable CD3 + T-cell neuroinflammatory profiles and TH + dopaminergic neuronal densities at the time of death. CD3 + T cells are indicated by arrows at 26.0× magnification. (**A**) Patient with longer disease duration and a lifetime cumulative levodopa dose of 1020 g, levodopa dose at death of 750 mg/day, and CD3 + T-cell density of 4.49 n/mm². (**B**) Corresponding SNc section from the same patient showing TH + neuron density of 2.97 n/mm². (**C**) Patient with shorter disease duration and a lifetime cumulative levodopa dose of 10 g who had discontinued levodopa one year prior to death. CD3 + T-cell density in this case was 4.58 n/mm². (**D**) Corresponding SNc section showing a TH + neuron density of 2.29 n/mm², similar to that observed in the patient with significantly higher levodopa exposure. These examples illustrate that cumulative levodopa exposure did not correspond to differences in SNc neuroinflammatory or dopaminergic neuronal profiles at end stage. Boxes overlaid on panels B and D indicate the approximate regions from which the high-magnification images in panels A and C were derived; however, the box size and position are illustrative only and not to scale with the magnified insets.

In PD patients, no significant associations were observed between levodopa exposure and neuronal density or neuroinflammation in the SNc or crus cerebri, after adjusting for age at death and sex (*p* > 0.17). Specifically, lifetime cumulative dose of levodopa (formulas 1 and 2), mean levodopa dose (formula 3), and levodopa dose at death were not associated with any neuropathological marker (Table 2; Figs. 1 and 2). Further analyses incorporating additional covariates – disease duration and HY stage – confirmed the absence of significant relationships (Table 2). Similarly, no significant differences in T cell infiltration or microglial density were observed between dyskinetic and non-dyskinetic PD patients (*p* > 0.14).

**Table 2:** Associations between Levodopa exposure, nigral neuron neurons or neuroinflammatory markers in PD patients. Values are β, 95% CI, and p-value.

	Formula 1 Lifetime levodopa dose		Formula 2 Lifetime levodopa dose		Formula 3 Mean levodopa dose		Daily levodopa dose at death	
Covariates: Age and Sex								
*n*	25	*p*-value	35	*p*-value	38	*p*-value	37	*p*-value
**LnTH count**	−2.330 × 10^−9^	0.989	−1.321 × 10^−7^	0.173	0.000	0.826	2.607 × 10^−5^	0.952
	−3.325 × 10^−7^; 3.371 × 10^−7^		−3.254 × 10^−7^;6.126 × 10^−8^		−0.001;0.001		−0.001;0.001	
**LnTH density**	−2.273 × 10^−9^	0.990	−7.782 × 10^−8^	0.455	0.000	0.823	−7.768 × 10^−5^	0.871
	−3.701 × 10^−7^;3.656 × 10^−7^		−2.877 × 10^−7^;1.320 × 10^−7^		−0.001;0.001		−0.001;0.001	
**LnCD3 count**	3.950 × 10^−8^	0.785	−9.871 × 10^−8^	0.267	7.451 × 10^−5^	0.859	5.712 × 10^−6^	0.988
	−2.584 × 10^−7^;3.374 × 10^−7^		−2.769 × 10^−7^;7.948 × 10^−8^		−0.001;0.001		−0.001;0.001	
**LnCD3 density**	−3.765 × 10^−10^	0.998	−4.152 × 10^−8^	0.606	2.057 × 10^−5^	0.957	0.000	0.766
	−2.615 × 10^−7^;2.608 × 10^−7^		−2.042 × 10^−7^;1.211 × 10^−7^		−0.001;0.001		−0.001;0.001	
**LnCD4 count**	−4.975 × 10^−7^	0.807	−1.267 × 10^−7^	0.254	0.000	0.804	8.726 × 10^−5^	0.854
	−4.688 × 10^−7^;3.693 × 10^−7^		−3.490 × 10^−7^;9.557 × 10^−8^		−0.001;0.001		−0.001;0.001	
**LnCD4 density**	−8.236 × 10^−7^	0.674	−7.779 × 10^−8^	0.466	0.000	0.711	−1.734 × 10^−5^	0.971
	−4.832 × 10^−7^;3.185 × 10^−7^		−2.929 × 10^−7^;1.373 × 10^−7^		−0.001;0.001		−0.001;0.001	
**LnCD8 count**	−2.416 × 10^−7^	0.912	−1.117 × 10^−7^	0.336	5.770 × 10^−5^	0.917	0.000	0.609
	−4.710 × 10^−7^;4.226 × 10^−7^		−3.450 × 10^−7^;1.215 × 10^−7^		−0.001;0.001		−0.001;0.001	
**LnCD8 density**	−7.477 × 10^−8^	0.726	−4.838 × 10^−8^	0.667	3.253 × 10^−5^	0.951	0.000	0.707
	−5.122 × 10^−7^;3.626 × 10^−7^		−2.756 × 10^−7^;1.788 × 10^−7^		−0.001;0.001		−0.001;0.001	
**LnIba1 SNc**	−1.306 × 10^−7^	0.613	5.616 × 10^−8^	0.667	0.000	0.711	0.000	0.827
	−6.602 × 10^−7^;3.989 × 10^−7^		−2.162 × 10^−7^;3.285 × 10^−7^		−0.001;0.002		−0.001;0.001	
**LnIba1 Crus Cerebri**	−1.060 × 10^−7^	0.503	1.689 × 10^−8^	0.837	0.000	0.416	0.000	0.498
	−4.298 × 10^−7^;2.177 × 10^−7^		−1.489 × 10^−7^;1.827 × 10^−7^		−0.001−0.001		−0.001;0.001	

**Covariates: Age**,** Sex**,** Hoehn and Yahr Stage**					**Covariates: Age**,** Sex**,** Hoehn and Yahr Stage**,** Disease Duration**			
**n**	**24**	**p-value**	**31**	**p-value**	**29**	**p-value**	**29**	**p-value**
**LnTH count**	−1.093 × 10^−7^	0.551	−6.047 × 10^−8^	0.582	8.664 × 10^−5^	0.873	0.000	0.599
	−4.868 × 10^−7^;2.682 × 10^−7^		−2.839 × 10^−7^;1.630 × 10^−7^		−0.001;0.001		−0.001;0.001	
**LnTH density**	−7.728 × 10^−8^	0.716	2.434 × 10^−9^	0.984	0.000	0.801	0.000	0.688
	−5.173 × 10^−7^;3.627 × 10^−7^		−2.498 × 10^−7^;2.547 × 10^−7^		−0.001;0.001		−0.001;0.001	
**LnCD3 count**	−2.216 × 10^−8^	0.892	−5.156 × 10^−8^	0.646	−1.728 × 10^−5^	0.974	−2.324 × 10^−5^	0.963
	−3.606 × 10^−7^;3.163 × 10^−7^		−2.800 × 10^−7^;1.769 × 10^−7^		−0.001;0.001		−0.001;0.001	
**LnCD3 density**	−7.499 × 10^−9^	0.961	−3.066 × 10^−9^	0.977	6.064 × 10^−5^	0.906	−4.487 × 10^−5^	0.926
	−3.285 × 10^−7^;3.135 × 10^−7^		−2.227 × 10^−7^;2.166 × 10^−7^		−0.001;0.001		−0.001;0.001	
**LnCD4 count**	−1.435 × 10^−7^	0.510	−2.758 × 10^−8^	0.843	0.000	0.598	0.000	0.845
	−5.917 × 10^−7^;3.048 × 10^−7^		−3.110 × 10^−7^;2.558 × 10^−7^		−0.001;0.001		−0.001;0.001	
**LnCD4 density**	−1.301 × 10^−7^	0.562	1.679 × 10^−8^	0.905	0.000	0.660	9.172 × 10^−5^	0.846
	−5.935 × 10^−7^;3.332 × 10^−7^		−2.698 × 10^−7^;3.034 × 10^−7^		−0.001;0.001		−0.001;0.001	
**LnCD8 count**	−1.291 × 10^−7^	0.623	−1.240 × 10^−7^	0.424	0.000	0.759	8.352 × 10^−5^	0.905
	−6.723 × 10^−7^;4.141 × 10^−7^		−4.382 × 10^−7^;1.903 × 10^−7^		−0.002;0.001		−0.001;0.002	
**LnCD8 density**	−1.155 × 10^−7^	0.662	−7.340 × 10^−8^	0.632	0.000	0.888	8.861 × 10^−5^	0.895
	−6.611 × 10^−7^;4.301 × 10^−7^		−3.855 × 10^−7^;2.387 × 10^−7^		−0.002;0.001		−0.001;0.001	
**LnIba1 SNc**	1.815 × 10^−7^	0.551	1.191 × 10^−7^	0.497	0.000	0.503	0.000	0.792
	−8.087 × 10^−7^;4.458 × 10^−7^		−2.369 × 10^−7^;4.752 × 10^−7^		−0.001;0.001		−0.001;0.001	
**LnIba1 Crus Cerebri**	−1.782 × 10^−7^	0.324	−1.499 × 10^−8^	0.891	0.000	0.516	0.000	0.628
	−5.471 × 10^−7^;1.907 × 10^−7^		−2.371 × 10^−7^;2.072 × 10^−7^		−0.001;0.001		−0.001;0.001	

Assessment of demographic and clinical factors showed that lifetime cumulative levodopa dose (formulas 1 and 2), mean levodopa dose and levodopa dose at death were positively correlated with disease duration (r=[0.38–0.75], *p* < 0.004) but not with age at death (r=[−0.47 to 0.18], *p* > 0.2). Levodopa doses did not significantly differ across HY stages (*p* > 0.78), and lifetime cumulative levodopa dose did not differ between men and women (*p* > 0.18). However, men had higher mean levodopa doses (median = 456 mg, IQR = 300 mg) compared to women (median = 200 mg, IQR = 517 mg, *p* = 0.047), as well as higher levodopa doses at death (median = 450 mg, IQR = 300 mg) compared to women (median = 200 mg, IQR = 575 mg, *p* = 0.037).

## PSP and MSA patients

After adjusting for disease duration, no significant associations were found between levodopa exposure and neuronal density, T cell infiltration or microglial density in either PSP or MSA (*p* > 0.012) (Supplementary Table 2). Additionally, age at death did not correlate lifetime cumulative levodopa dose (formulas 1 and 2), mean levodopa dose or levodopa dose at death (*p* > 0.026), and no sex-based differences were observed in either disease group (*p* > 0.5).

## Discussion

The primary objective of this study was to assess whether chronic levodopa treatment influences nigral neuronal survival or neuroinflammatory activity in PD, PSP and MSA. Our findings demonstrate that cumulative levodopa exposure is not associated with neuroinflammatory markers or neuronal loss in the SNc across these disorders. These findings suggest that chronic levodopa use is not associated with sustained nigral neuroinflammation or dopaminergic cell loss at end-stage disease in PD, PSP, or MSA. While these results offer indirect support for the safety of long-term levodopa treatment, they do not exclude the possibility of earlier or transient neuroinflammatory changes during the disease course.

Neuroinflammation has emerged as a central mechanism in the pathogenesis of parkinsonian disorders^17,18,25^. However, the interplay between dopaminergic therapy and neuroinflammation has remained poorly defined. Preclinical models of PD have suggested that chronic levodopa treatment may amplify neuroinflammatory signaling, particularly in the context of levodopa-induced dyskinesias. These experimental studies have reported microglial activation and increased expression of pro-inflammatory cytokines in the basal ganglia following prolonged levodopa administration, with some evidence suggesting that immunomodulatory strategies can mitigate the development of dyskinesias^26–28^. Despite these experimental observations, the present postmortem findings did not reveal significant differences in neuroinflammatory markers between dyskinetic and non-dyskinetic PD patients. However, it is important to acknowledge that only four levodopa-treated PD patients in our cohort had a documented history of dyskinesia. Given the high prevalence of dyskinesia in long-term levodopa-treated PD patients^29^, this low number possibly reflects underreporting in clinical records rather than a true absence of the phenomenon. Nonetheless, our broader analysis demonstrated no correlation between lifetime levodopa exposure and T cell infiltration or microglial density across PD, MSA, and PSP. These findings suggest that levodopa neither drives nor suppresses chronic nigral neuroinflammation in parkinsonian disorders.

A critical consideration in interpreting our results is that the analyses were conducted at the end stage of disease. While we cannot exclude the possibility that levodopa induces transient neuroinflammatory responses earlier in the disease course, any such effects do not appear to translate into sustained neuroinflammation. This is consistent with the finding that cumulative levodopa exposure was not associated with reduced TH+ neuronal counts in the SNc, reinforcing the view that levodopa does not contribute to more aggressive dopaminergic neurodegeneration. These results add to a growing body of evidence from clinicopathological studies, neuroimaging research, and clinical trials refuting the notion of levodopa-induced neurotoxicity^12,30–32^. Our findings not only replicate these conclusions in an independent PD cohort but also extend them to atypical parkinsonian syndromes, demonstrating the levodopa does not impact neuronal survival in PSP or MSA either.

Although the mean cumulative levodopa dose in our PD cohort (0.68 kg) was notably lower than that reported in the postmortem study by Parkkinen et al. (3.3. kg)^12^, this discrepancy likely reflects differences in patient demographics and disease characteristics. The average disease duration in our study was 9.0 years compared to 15.3 years in Parkkinen’s cohort, and the mean age at onset was substantially higher (72 vs. 60 years). Since younger patients are typically treated with higher daily levodopa doses^33^ and experience longer disease courses, these factors likely explain the observed dose disparity. Importantly, our results demonstrate that even in an older PD population – arguably more vulnerable to treatment-related cellular stress – there is no evidence of levodopa-associated nigral inflammation or neuronal loss. This perspective increases the generalizability of prior findings by providing reassurance regarding levodopa safety in geriatric PD populations.

While our study provides insights into the relationship between neuroinflammation and levodopa use, several limitations should be considered. First, the sample sizes for PSP and MSA cohorts were relatively small, which may limit the statistical power to detect subtle effects of levodopa exposure in these populations. However, the large interindividual variability in total levodopa doses within PSP and MSA groups, without corresponding differences in neuropathological outcomes, strengthens the robustness of the conclusions despite the smaller cohort sizes. Additionally, clinical medication records were not always complete, and missing data in some cases may have influenced cumulative levodopa exposure estimates. As a result, any potential association between dyskinesia severity and neuroinflammatory markers may not have been adequately captured in the analysis due to possible underreporting. The observed prevalence of dyskinesia in the cohort was notably low, which may reflect underreporting or inaccurate documentation. Alternatively, it is also possible that dyskinesia was attenuated by clinical adjustment strategy, such as gradual reduction in levodopa dosage in response to emerging motor complications. Given that levodopa dosage adjustments over time are influenced by disease progression, treatment response and side effect profiles, accurately reconstructing lifetime levodopa exposure remains inherently challenging. An important limitation is the inherent cross-sectional nature of postmortem studies, which precludes assessment of dynamic or transient neuroinflammatory processes that may have occurred earlier in the disease course. It remains possible that levodopa exerts short-term immunomodulatory effects that are not sustained into late-stage disease. Additionally, while cumulative dose provides a clinically relevant measure of total exposure, it may not fully capture the temporal dynamics of levodopa’s pharmacological impact on inflammation, which may fluctuate in response to multiple factors, including disease activity and comorbidities. Future studies incorporating longitudinal in vivo assessments of neuroinflammation, such as PET imaging with microglial tracers, may provide a dynamic perspective on the interaction between levodopa therapy and neuroimmune mechanisms. Furthermore, while the current study focused on cellular immune markers such as T lymphocyte infiltration and microglial density, we did not include direct molecular markers of inflammation, such as pro-inflammatory cytokines (e.g., IL-1β, IL-6, TNFα). These cytokines represent key mediators of neuroinflammation and may provide additional mechanistic insights. Future studies incorporating multiplex cytokine quantification, transcriptomic profiling, or PET imaging with microglial tracers could complement the current histological data and offer a more comprehensive understanding of levodopa’s potential immunomodulatory effects.

In conclusion, our findings indicate that chronic levodopa use does not influence nigral neuronal survival or neuroinflammatory activity in PD, PSP, or MSA. These results reinforce the well-established role of levodopa as an effective symptomatic treatment without significant disease-modifying properties and provide reassurance regarding its long-term safety, alleviating concerns about potential inflammatory neurotoxic properties.

## Supplementary Information

Below is the link to the electronic supplementary material.

Supplementary Material 1

Supplementary Material 2

## Data Availability

The data supporting the findings of this study are available from the corresponding author upon reasonable request.
