# Supplementary material for: Levodopa exposure and nigral neuroinflammation in parkinsonian disorders: A postmortem study of 63 cases
Source: Sci Rep. 2025 Nov 11;15:39516. doi: 10.1038/s41598-025-23376-2 (PMC12606330; doi:10.1038/s41598-025-23376-2)
Supplement: Supplementary file 1 — Supplementary Material 1 [file 41598_2025_23376_MOESM1_ESM.docx]

**Supplementary Table 1.** Associations between different levodopa formulas across all groups (PD, PSP, MSA, n=63). Values are Spearman correlation coefficient (p-value).

|  | **Formula 1** | **Formula 2** | **Formula 3** | **Daily levodopa dose at death** |
| --- | --- | --- | --- | --- |
| **Formula 1** | - | 0.88  <0.001 | 0.76  <0.001 | 0.80  <0.001 |
| **Formula 2** | 0.88  <0.001 | - | 0.79  <0.001 | 0.78  <0.001 |
| **Formula 3** | 0.76  <0.001 | 0.79  <0.001 | - | 0.95  <0.001 |
| **Daily levodopa dose at death** | 0.80  <0.001 | 0.78  <0.001 | 0.95  <0.001 | - |
