# Supplementary material for: Levodopa exposure and nigral neuroinflammation in parkinsonian disorders: A postmortem study of 63 cases
Source: Sci Rep. 2025 Nov 11;15:39516. doi: 10.1038/s41598-025-23376-2 (PMC12606330; doi:10.1038/s41598-025-23376-2)
Supplement: Supplementary file 2 — Supplementary Material 2 [file 41598_2025_23376_MOESM2_ESM.docx]

**Supplementary Table 2.** Associations of between levodopa exposure, nigral neuron neurons or neuroinflammatory markers in MSA and PSP patients. Values are Spearman Partial correlation coefficient and p-value.

|  | **Formula 1**  **Lifetime levodopa dose (MSA)** | **Formula 2**  **Lifetime levodopa (MSA)** | **Formula 3**  **Mean levodopa dose (MSA)** | **Daily levodopa dose at death (MSA)** | **Formula 1**  **Lifetime levodopa dose (PSP)** | **Formula 2**  **Lifetime levodopa (PSP)** | **Formula 3**  **Mean levodopa dose (PSP)** | **Daily levodopa dose at death (PSP)** |
| --- | --- | --- | --- | --- | --- | --- | --- | --- |
| **Controlling factor: disease duration** | | | | | | | | |
| **n** | **12** | **12** | **12** | **12** | **13** | **13** | **13** | **13** |
| **LnTH count** | -0.24  0.471 | -0.25  0.453 | -0.25  0.463 | -0.28  0.413 | 0.26  0.424 | 0.10  0.756 | -0.05  0.876 | -0.08  0.807 |
| **LnTH density** | -0.15  0.665 | -0.17  0.608 | -0.23  0.492 | -0.26  0.436 | 0.14  0.669 | -0.05  0.844 | -0.12  0.721 | -0.14  0.656 |
| **LnCD3 count** | -0.14  0.690 | -0.14  0.692 | -0.05  0.878 | -0.04  0.915 | -0.14  0.666 | -0.17  0.599 | 0.10  0.748 | -0.11  0.729 |
| **LnCD3 density** | -0.16  0.736 | -0.06  0.873 | 0.01  0.968 | 0.02  0.958 | -0.42  0.170 | -0.45  0.147 | -0.19  0.556 | -0.34  0.278 |
| **LnCD4 count** | -0.00  0.992 | -0.08  0.806 | 0.05  0.877 | 0.04  0.918 | 0.67  0.018 | 0.69  0.013 | 0.49  0.104 | 0.53  0.078 |
| **LnCD4 density** | 0.09  0.793 | 0.04  0.915 | 0.16  0.648 | 0.13  0.703 | 0.39  0.208 | 0.40  0.190 | 0.32  0.306 | 0.36  0.251 |
| **LnCD8 count** | 0.22  0.524 | 0.27  0.414 | 0.30  0.370 | 0.31  0.362 | 0.33  0.299 | 0.20  0.537 | 0.04  0.892 | -0.18  0.582 |
| **LnCD8 density** | 0.27  0.418 | 0.32  0.345 | 0.33  0.328 | 0.32  0.333 | 0.20  0.529 | 0.16  0.722 | -0.01  0.980 | -0.22  0.491 |
| **LnIba1 SNc** | -0.02  0.949 | 0.10  0.767 | 0.01  0.975 | -0.02  0.960 | 0.27  0.395 | 0.28  0.387 | 0.13  0.688 | 0.06  0.848 |
| **LnIba1 Crus Cerebri** | 0.10  0.764 | 0.06  0.872 | -0.05  0.893 | -0.07  0.838 | 0.26  0.419 | 0.28  0.376 | 0.21  0.511 | 0.22  0.485 |
